# The IVF Outcome Counseling Based on the Model Combining DHEAS and Age in Patients with Low AMH Prior to the First Cycle of GnRH Antagonist Protocol of Ovarian Stimulation

**DOI:** 10.1155/2013/637919

**Published:** 2013-02-24

**Authors:** Miro Šimun Alebić, Nataša Stojanović, Marta Žuvić-Butorac

**Affiliations:** ^1^Department of Human Reproduction, Merkur Teaching Hospital, 10 000 Zagreb, Croatia; ^2^Institute of Clinical Chemistry and Laboratory Medicine, Merkur Teaching Hospital, 10 000 Zagreb, Croatia; ^3^Department of Biotechnology, University of Rijeka, 51 000 Rijeka, Croatia

## Abstract

*Objective*. To investigate the endocrine and/or clinical characteristics of women with low anti-Müllerian hormone (AMH) that could improve the accuracy of IVF outcome prediction based on the female age alone prior to the first GnRH antagonist IVF cycle. *Methods*. Medical records of 129 patients with low AMH level (<6.5 pmol/L) who underwent their first GnRH antagonist ovarian stimulation protocol for IVF/ICSI were retrospectively analyzed. The main outcome measure was the area under the ROC curve (AUC-ROC) for the models combining age and other potential predictive factors for the clinical pregnancy. *Results*. Clinical pregnancy rate (CPR) per initiated cycles was 11.6%. For the prediction of clinical pregnancy, DHEAS and age showed AUC-ROC of 0.726 (95%CI 0.641–0.801) and 0.662 (95%CI 0.573–0.743), respectively (*P* = 0.522). The predictive accuracy of the model combining age and DHEAS (AUC-ROC 0.796; 95%CI 0.716–0.862) was significantly higher compared to that of age alone (*P* = 0.013). In patients <37.5 years with DHEAS >5.7 pmol/L, 60% (9/15) of all pregnancies were achieved with CPR of 37.5%. *Conclusions*. DHEAS appears to be predictive for clinical pregnancy in younger women (<37.5 years) with low AMH after the first GnRH antagonist IVF cycle. Therefore, DHEAS-age model could refine the pretreatment counseling on pregnancy prospects following IVF.

## 1. Introduction

Counseling infertile couples about chances of IVF success is one of the most demanding clinician's tasks. Since the number of oocytes retrieved and fertilized is found to be of the highest predictive value in pregnancy prediction models based on intermediate results of IVF treatment, patients in whom low number of oocytes were retrieved after controlled ovarian stimulation for IVF could generally considered as having poor pregnancy prospects [1–4]. Obviously, data on previous ovarian stimulations are not available before the first IVF cycle, so clinician should be able firstly to identify the patients at risk of poor response in order to optimize the stimulation dose regimen and secondly to counsel them on probability of pregnancy as accurately as possible by using baseline patient characteristics.

Serum anti-Müllerian hormone (AMH) level is considered to be a valuable quantitative marker of ovarian reserve [5–8]. It is predictive of poor response to ovarian stimulation with exogenous gonadotrophins, but there is no convincing evidence supporting the value of AMH measurement in predicting pregnancy and live birth after IVF yet [7, 9–11]. However, protocols with dose adjustment according to AMH were demonstrated to improve ovarian response and treatment outcome [3, 12].

Female age is the most important patient characteristic in determining prospects of IVF success [1], although value of age as a single predictor of pregnancy was not found to be optimal [7].

The model combining AMH and age was recently demonstrated as having the moderate value in prediction of live birth after IVF suggesting that in low AMH category, younger women are more likely to become pregnant then women with advanced age [10]. 

Other investigated patient characteristics either failed to demonstrate any added value to female age in predicting pregnancy after IVF or their values were not clearly established yet [4, 7].

The rationale for this study was to improve the prediction of pregnancy chances at initial IVF counseling in patients expected to poorly respond on ovarian stimulation based on their AMH levels, since poor responders are not homogeneous group regarding the pregnancy prospects [4]. 

Accordingly, the objectives of this study were firstly to determine whether any of investigated clinical and biochemical characteristic(s) add(s) value to female age in predicting clinical pregnancy prior to the first GnRH antagonist protocol of ovarian stimulation for IVF/ICSI in patients with low AMH values (<6.5 pmol/L), and secondly to develop an easy-to-use counseling approach that would more accurately than the model based on female age alone differentiate patients with low AMH who show favorable prognosis for pregnancy from those with little chances for treatment success.

## 2. Materials and Methods

### 2.1. Study Population

In this study, medical records on IVF treatments and laboratory test results derived from the electronic database in Department of Human Reproduction of Merkur Teaching Hospital (Zagreb, Croatia) were analyzed retrospectively. All data were prospectively collected and recorded by authorized staff of the department between October 2010 and February 2012. The IVF cycles included in the analysis met all of the following criteria: (1) null gravidity, (2) normal uterus and uterine cavity, (3) no history of pelvic disease or surgery, (4) no history of the use of medications that could interfere with basal hormone status, (5) sperm count of, at least, 1 × 10^6^/mL, (6) first IVF/ICSI cycle, (7) AMH and other laboratory tests values obtained within three months preceding controlled ovarian stimulation, (8) serum AMH concentration <6.5 pmol/L, (9) a fixed dose of 300 I.U. hMG administered daily from the day 3 in the GnRH antagonist protocol of ovarian stimulation with an oral contraceptive (OC) pretreatment. 

It has been previously demonstrated that serum AMH concentrations <5 pmol/L identify women who demonstrate poor ovarian response and reduced clinical pregnancy rate (CPR) independent of treatment strategy and age [3, 6]. This cut-off value was determined using DSL MIS/AMH ELISA (reference DSL-10-14400; DSL, Webster, USA). In our laboratory serum AMH concentrations were measured using AMH Gen II ELISA (Beckman Coulter, Inc, Brea, USA). Although method comparison has shown a good agreement between the results obtained using DSL AMH/MIS and AMH Gen II, the serum AMH values obtained by the AMH Gen II assay were approximately 40% higher [13]. Therefore, in the present study, the AMH threshold value for the poor ovarian response was arbitrarily set at the higher level (6.5 pmol/L).

All IVF patients gave an informed consent at initial consultation for their data to be used for clinical research, statistical reports, and/or educational purposes provided that their identity remains protected. This study was approved by the institutional Ethics Committee.

### 2.2. Stimulation Protocol

A standard GnRH antagonist protocol was used for controlled ovarian stimulation. After OC pretreatment for 21–30 days, which was used for scheduling purposes, ovarian stimulation was commenced on cycle day 3 with a daily dose of 300 I.U. of hMG (Menopur; Ferring GmbH, Kiel, Germany) subcutaneously for five days, if the presence of ovarian cysts was excluded by TV-US. According to routine clinical practice, follicular responses were monitored with serum *E*_2_ measurements and by transvaginal ultrasonography (TV-US) to assess the follicular growth and to define proper timing for triggering final oocyte maturation. 

On the cycle day 8, the TV-US was performed and the *E*_2_ concentration was determined. If significant increase in *E*_2_ concentration occurred and at least one follicle >11 mm in diameter was visualized on TV-US, the stimulation was continued at the same hMG dose, and administration of the GnRH antagonist (Cetrotide; Baxter Oncology GmbH, Frankfurt, Germany) was started at a daily dose of 0.25 mg (subcutaneously) to be continued until the morning of the day of hCG administration. Alternatively, the stimulation was cancelled. Final oocyte maturation was induced with 10 000 I.U. of hCG (Brevactid 5000 I.E., Ferring GmbH, Kiel, Germany), i.m., when at least one follicle >17 mm in diameter was present on TV-US. Oocytes were retrieved 36 hours after hCG administration by TV-US guided aspiration. All patients in whom the oocytes were retrieved received 1500 I.U. of hCG for luteal phase support on the day of oocyte retrieval. Fertilization of oocytes was performed by IVF or ICSI in compliance with Croatian law regulating medically assisted reproduction. This law was in force from September 2009 until July 2012 and imposed that no more than three oocytes can be fertilized at one time and all embryos obtained must be transferred simultaneously. Accordingly, if ≥3 oocytes were retrieved, 3 oocytes were selected for fertilization by standard procedure and the remaining good-quality oocytes were frozen. All embryos obtained were transferred under ultrasonographic guidance two or three days after oocyte retrieval. Luteal phase support with progesterone capsules (Utrogestan 100 mg; Laboratories Besins International, Montrouge, France), 200 mg three times a day, was given intravaginally from the day of oocyte retrieval until the day of serum hCG determination (18 ± 1 day after oocyte retrieval). If pregnancy was confirmed by determination of serum hCG, luteal phase support with Utrogestan 200 mg continued until 12 weeks of gestational age. Clinical pregnancy was confirmed if fetal cardiac activity was observed at TV-US 28 ± 1 day after oocyte retrieval.

### 2.3. Laboratory Analysis

Blood samples for the hormone measurements were taken during the early follicular phase of menstrual cycle (i.e., cycle days 3–5 of a spontaneous menstrual cycle or a withdrawal bleeding induced by 100 mg of micronized progesterone vaginally tid for 10 days) between 8:00 and 10:00 hours after an overnight fast. Serum concentrations of DHEAS, *E*_2_, FSH, hCG, LH, PRL and T were determined by chemiluminescent immunoassays (Beckman Coulter, Inc., Fullerton, USA). The limit of detection, within- and between-assay coefficients of variation (CVs) were as follows: 0.05 *μ*mol/L, 2.5% and 2.1% for DHEAS; 73 pmol/L, 6.0% and 3.4% for *E*_2_; 0.2 IU/L, 2.9% and 3.4% for FSH; 0.5 IU/L, 1.6% and 2.6% for hCG; 0.2 IU/L, 4.2% and 2.7% for LH; 5.3 mIU/L, 1.9% and 1.1% for PRL, 0.35 nmol/L, 3.6% and 2.3% for T. TSH concentration was measured by using the ADVIA Centaur XP immunoassay system (Siemens Healthcare Diagnostics, Inc., Tarrytown, USA). The limit of detection, within- and between-assay CVs were 0.001 mIU/L, 2% and 4% for TSH. The total precision of the DHEAS, *E*_2_, FSH, hCG, LH, PRL, T, and TSH measurements was as follows: 3%, 6%, 3.9%, 4.2, 5%, 2.4%, 3.8%, and 3.2% (calculated by using guidelines described in CLSI document EP15-A2) [14]. Serum AMH concentration was determined using AMH Gen II ELISA (Beckman Coulter, Inc., Brea, USA). For AMH, the limit of quantification is 1.14 pmol/L and the limit of detection is 0.56 pmol/L as specified by the manufacturer. Within- and between-assay CVs were 2.5% and 1.5%, respectively. Total precision demonstrated in our laboratory was 3.1% [14]. All biochemistry analyses were performed in the laboratory accredited according to EN ISO 15189.

### 2.4. Clinical Investigation

Anthropometric measurements and transvaginal ultrasound scanning were performed the same day as blood drawing for the hormone analysis. The number of follicles measuring 2–9 mm in diameter (AFC) in each ovary was assessed by a single investigator (M.Š.A.) using a two-dimensional transvaginal probe 5–7 MHz (Toshiba, Nemio, Japan). 

### 2.5. Statistical Methods

Medcalc Software version 12.3.0 (Mariakerke, Belgium) was used for statistical analysis. Baseline patient characteristics, treatment, and IVF outcomes data of pregnant and nonpregnant patients were compared using Mann-Whitney test. Univariate and multivariate logistic regression analyses were used to investigate the potential association between independent variables (age, body mass index (BMI), AFC, AMH, DHEAS, *E*_2_, FSH, LH, T, PRL, TSH) and the dependent variable (clinical pregnancy). The receiver operating characteristic (ROC) curves were plotted firstly to determine the discriminative power of the tested variables using area under the receiver operating characteristic curve (AUC-ROC), and secondly to determine the cut-off values with the optimal performances for discrimination between pregnancy and non-pregnancy. The Kruskal-Wallis test with post-hoc analysis and the Fisher's exact test were used for comparison of the IVF outcomes between the groups stratified using the cut-off values of the variables identified as predictive of the clinical pregnancy. The AUC-ROC comparison was used to compare predictive performances of the multivariate model and the age-alone model. *P* value of <0.05 was considered as statistically significant. Due to rigorous inclusion criteria and limited number of events in the original dataset, developed counseling approach was internally validated on the 1000 bootstrap replications of the original sample. The classification and reclassification of patients according to their pregnancy chances were performed based on the age-alone model and the multivariate model, respectively, in order to assess the clinical usefulness of the approach [15]. 

## 3. Results

Medical records of 803 IVF cycles were searched using inclusion criteria and 129 cycles were selected for the statistical analysis. There were 7% (9/129) cancelled cycles, 9.3% (12/129) cycles in which no oocytes were retrieved, and 19.4% (25/129) cycles ended by a fertilization failure. In total, 64.3% (83/129) of initiated cycles were followed by embryo transfer. Implantation rate (total number of viable embryos × 100/total number of embryos transferred) was 12.8% (20/156) and clinical pregnancy rate per initiated cycle (CPR) was 11.6% (15/129). Two clinical pregnancies were recorded in patients with AMH concentration below the limit of quantification (<1.14 pmol/L). When baseline patient characteristics were compared, statistically significant differences in age and DHEAS concentration between pregnant and nonpregnant group were found (*P* = 0.043 and *P* = 0.005, resp.). All other baseline clinical and biochemical characteristics as well as the treatment data were similar in both groups (Table 1).

The results of univariate and multivariate logistic regression analysis are shown in Table 2. The univariate logistic regression analysis revealed DHEAS as the best single predictor of clinical pregnancy (AUC-ROC 0.726, 95%  CI 0.641–0.801). However, when AUC-ROC of DHEAS was compared to that of the age, as the second best single predictor, the predictive power of DHEAS was not found to be substantially and significantly different (*P* = 0.522). The predictive potential for clinical pregnancy of all other tested variables was not demonstrated. 

**Figure 1:**
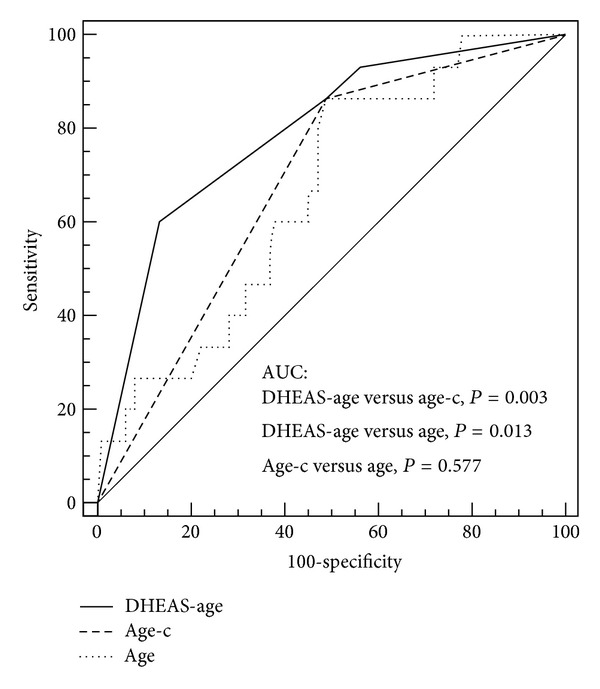
Comparison of area under the receiever operating characteristics curve (AUC-ROC) of the multivariate model for clinical pregnancy prediction combining DHEAS and female age (DHAES-age model) and the univariate models based on age as continuous or categorical variable. DHEAS-age = multivariate categorical model using DHEAS as categorical variable with cut-off value of 5.7 *μ*mol/L and age as categorical variable with cut-off value of 37.5 years; age-c = univariate model using age as categorical variable with cut-off value of 37.5 years; age = univariate model using age as continuous variable; *P* value < 0.05 is considered statistically significant.

The cut-off values of 37.5 years for age (odds ratio (OR) 6.7; 95%  CI 1.5–31.2) and 5.7 *μ*mol/L for DHEAS (OR 7.9; 95%  CI 2.5–25.4) were derived from ROC curve analysis as having optimal performances in discrimination between pregnancy and non-pregnancy. The subsequent multivariate logistic analysis using DHEAS and age as continuous variables eliminated the age from the model for the pregnancy prediction. Therefore, the categorical model for clinical pregnancy prediction based on the cutoffs for age and DHEAS (DHEAS-age model) was developed. The better predictive performance of DHEAS-age model compared to the age-alone model was demonstrated by the AUC-ROC comparison (Table 2, Figure 1).

In order to internally validate the clinical value of the DHEAS-age model, the patients were grouped according to the selected cut-off values for the age and DHEAS (Table 3). In the <37.5 years age category, DHEAS discriminated well between pregnancy and non-pregnancy (AUC 0.712; 95%  CI 0.591–0.815; *P* = 0.004). 

As shown in Table 3, the observed CPR was high in the <37.5 years and DHEAS > 5.7 *μ*mol/L cohort (Group 1, *n* = 24) and significantly different from the CPR of the age-matched group of patients with DHEAS ≤ 5.7 *μ*mol/L (Group 2, *n* = 45), 37.5% versus 8.9% (*P* < 0.05). Furthermore, 60% (9/15) of all pregnancies were achieved in Group 1. The number of oocytes retrieved and the number of embryos transferred did not differ between these two groups. Significant between-group differences in other baseline patient characteristics were not found in <37.5 years age category of the patients, except in T levels. 

Within the ≥37.5 years age category, patients with DHEAS > 5.7 *μ*mol/L (Group 3, *n* = 9) had higher CPR than patients with DHEA-S ≤ 5.7 *μ*mol/L (Group 4, *n* = 51). However, the observed difference did not reached the level of significance. In this age-category, the data on DHEAS levels did not provide an additional information regarding the pregnancy prospects (AUC-ROC 0.681; *P* = 0.471). 

Notably, the significant difference was not found when CPR in Group 2 was compared with CPR in Group 4 (8.9% versus 2%; *P* = 0.291). As depicted in Table 3, there was no substantial difference in CPR between these two groups despite the significant difference in age, the number of the oocytes retrieved, and the number oocytes eligible for fertilization.

The AUC-ROC of the DHEAS-age model after correction for overoptimism was 0.790 (95%  CI 0.710–0.857). The predicted over observed CPRs in bootstrapped samples were 0.98, 1.03, 1.07, 0.83 for each group, demonstrating a good calibration of the model.

After DHEAS-age model was applied on the study population, 15% (9/60) of those patients previously classified by age-alone model as having <5% chance for pregnancy and 65% (45/69) of those classified as having >20% chance were reclassified in the new category with the pregnancy probability of *≈*10%.

## 4. Discussion

It is generally recognized that the poor responders have lower pregnancy rates than normal responders, regardless of definition used for the poor response to ovarian stimulation [4, 16, 17]. In the present study, AMH level (cut-off value of 6.5 pmol/L) was used for identification of the patients at risk for the poor response.

In overall study population the CPR following the ovarian stimulation for IVF according to GnRH antagonist protocol was 11.6%, which is in accordance with previous reports on reduced pregnancy prospects in women with diminished ovarian reserve [3, 6, 18]. 

However, our study results failed to demonstrate the association between the AMH levels and IVF outcomes. The AMH levels in the pregnant and nonpregnant women did not differ (Table 1) and two pregnancies were recorded in women with negligible AMH levels (<1.14 pmol/L) supporting previous suggestions that AMH is not suitable to be used as a single predictor in the pregnancy prospect assessment [7, 18, 19]. 

The important role of age in the pregnancy prospects was confirmed in our study population too [7, 18]. The age cut-off value of 37.5 years demonstrated the best performance for the discrimination of the patients with diminished ovarian reserve who will or will not become pregnant after assisted conception. The patients with low AMH and ≤37.5 years of age had higher CPR then their older counterparts, 23.2% versus 3.5% (*P* < 0.001) which also confirms recently reported association between age and IVF outcomes within the given AMH category [10].

Among all the other clinical and biochemical characteristics tested, DHEAS was found to be the best single predictor of clinical pregnancy in our study population. DHEAS is a sulfated metabolite of DHEA which is involved in ovarian steroidogenesis as a hormone precursor, and can be partially metabolized into active androgens and estrogens in peripheral tissues [20]. Levels of DHEA(S) decline linearly and systematically with age [21, 22] and epidemiological studies suggest that DHEA(S) may have beneficial effect on age-related conditions [23]. According to the recent concept of ovarian ageing, DHEA supplementation may beneficially affect aging of ovarian environments and thus improve a oocyte/embryo quality [24]. 

In this study, the overall predictive power of the DHEAS-age model was significantly better in predicting pregnancy compared to the model based on female age alone (Table 2). However, DHEAS levels seem to be related to the chances for pregnancy only in the <37.5 year age category. In this age category, the differences in other baseline patient characteristics between the two groups (Group 1 and Group 2) differing in the DHEAS levels were not observed, with exception of T which could be attributed to the enhanced androgenic conversion of the higher amount of DHEA (Table 3). Together with the lack of differences in the number of oocytes retrieved and embryos transferred in these women, the observed differences in CPR and implantation rate could be explained by the possible influence of DHEA(S) on the oocyte quality rather than quantity as recently proposed [24]. 

The observed difference in CPRs between groups differing in the DHEAS levels within ≥37.5 years age category failed to reach the level of significance which could be attributed to the small number of patients with higher DHEAS levels in this age category (Table 3). However, similar CPR in patients <37.5 years and DHEAS ≤ 5.7 *μ*mol/L (Group 2) and patients ≥37.5 years and DHEAS ≤ 5.7 *μ*mol/L (Group 4) suggests that the potential effect of DHEAS deficiency on the oocyte quality reduces the pregnancy chances in younger patients to the level inherent to the older age categories (Table 3).

Our study results support outcomes of previous studies suggesting the continuation of pursuit for the patient characteristics potentially associated with IVF outcome success and the need for development of the more accurate predictive models that will refine patient counseling approach as the accuracy of currently used predictors of pregnancy after IVF is not optimal [4, 25]. 

If age-alone model for pregnancy prospects had been applied at initial patient counseling and 37.5 years used as the cut-off value for the pregnancy prediction, 53.5% (69/129) and 46.5% (60/129) of couples from our study population should had been counseled as to have >20% and <5% chances for pregnancy, respectively. Here, presented data suggest that the information on the DHEAS level might be helpful in pregnancy prospects assessment prior to the first IVF treatment with GnRH antagonist ovarian stimulation protocol at least in women <37.5 years with low AMH. Patients <37.5 years with higher DHEAS levels could, accordingly, be reassured as having their chances for pregnancy similar to that of general IVF population. On the other hand, patients with lower DHEAS from the same age category could be counseled as having their pregnancy prospects similar to that of patients' ≥37.5 years. Using this approach, 42% of patients could be counseled more accurately compared to the prediction model that relies on the female age only.

The main limitation of this study is the small number of patients that is mainly a consequence of the rigorous inclusion criteria as only patients who underwent their first IVF cycle and were treated with the same initial dose of gonadotrophins in GnRH antagonist protocol of ovarian stimulation were selected for the analysis. Further limitations are the DHEAS cut-off specificity for the applied DHEAS immunoassay and particular Croatian legislation setting which both prevent universal application of here presented counseling approach for expected poor responders. 

In summary, the information on DHEAS levels could improve clinician's ability to counsel the couples more accurately about the probabilities for successful IVF treatment outcome in women with low AMH who were younger than 37.5 years. Further studies, prospectively addressing this specific issue on the large patient population in different settings are needed to evaluate these findings. 

## Figures and Tables

**Table 1:** Baseline patient and treatment characteristics of studied IVF cycles resulting in clinical pregnancy and nonpregnancy.

Variable	Overall group	Nonpregnant	Pregnant	*P* value^a^
	(*n* = 129)	(*n* = 114)	(*n* = 15)	
Age (y)	37.0 ± 3.7	37.3 ± 3.7	35.0 ± 3.6	0.043
BMI (kg/m^2^)	23.9 ± 3.8	24.0 ± 3.9	23.5 ± 2.0	0.976
AFC	6.6 ± 3.7	6.4 ± 3.6	8.1 ± 4.3	0.134
AMH (pmol/L)	3.0 ± 1.7	3.0 ± 1.7	3.5 ± 1.9	0.246
DHEAS (*μ*mol/L)	4.5 ± 1.9	4.3 ± 1.8	5.9 ± 2.2	0.005
FSH (IU/L)	9.7 ± 6.7	9.8 ± 7.1	9.1 ± 3.2	0.752
LH (IU/L)	4.6 ± 2.2	4.7 ± 2.3	4.5 ± 1.6	0.755
*E*_2_ (pmol/L)	223 ± 123	223 ± 123	224 ± 102	0.823
*T* (nmol/L)	1.4 ± 0.7	1.4 ± 0.6	2.3 ± 1.0	0.273
Duration of stimulation (d)	10.3 ± 1.9	10.3 ± 1.9	10.5 ± 0.8	0.688
Total gonadotrophins used (IU)	3095.5 ± 553.6	3089.5 ± 582.4	3140 ± 250.1	0.741
Number of ICSI cycles, % (*n*/*N*)	75.0 (81/108)	75.3 (70/93)	73.3 (11/15)	0.540

Note: values are presented as mean ± SD. AFC: antral follicle count; AMH: anti-Müllerian hormone; BMI: body mass index. Conversion formulas for the AMH, DHEAS, *E*_2_, and *T* values in mass units are as follows: ng/mL = pmol/L: 7.14, *μ*g/dL × 0.027 = *μ*mol/L, pg/mL × 3.67 = pmol/L, and ng/dL × 0.0347 = mmol/L.

^a^pregnant versus nonpregnant.

**Table 2:** Univariate and multivariate logistic regression analysis of variables studied for the prediction of clinical pregnancy in patients with low AMH and area under the receiver operating characteristic curves (AUC-ROC) analysis.

Variable	*R* ^2^	*P* value	AUC-ROC	95% CI
Univariate continuous models				
DHEAS	0.466	0.004	0.726	0.641–0.801
Age	−0.173	0.031	0.662	0.573–0.743
AFC	0.115	0.104	0.619	0.529–0.703
AMH	0.172	0.260	0.592	0.502–0.678
BMI	−0.038	0.626	0.502	0.413–0.592
*E*_2_	0.086	0.969	0.518	0.428–0.607
FSH	−0.020	0.661	0.525	0.435–0.614
LH	−0.024	0.848	0.525	0.435–0.613
*T*	0.473	0.234	0.587	0.497–0.673
PRL	−0.008	0.843	0.501	0.412–0.590
TSH	−0.044	0.747	0.544	0.454–0.632
Univariate categorical model				
Age-c	−1.907	0.015	0.688	0.600–0.766
Multivariate categorical model				
DHEAS-age	−1.167	0.001	0.796	0.716–0.862

Note: *R* ^2^: regression coefficient; *P*: *P* value for *R* ^2^; CI: confidence interval; AFC: antral follicle count; AMH: anti-M$\ddot{\text{u}}$llerian hormone; BMI: body mass index; age-c: univariate categorical model using age as categorical variable with cut-off value of 37.5 years; DHEAS-age: multivariate categorical model using DHEAS as categorical variable with cut-off value of 5.7 μmol/L and age as categorical variable with cut-off value of 37.5 years.

**Table 3:** Baseline patient characteristics, treatment variables, and IVF outcomes of the patients grouped according to the cut-off values for female age and DHEAS level predictive of clinical pregnancy (cutoffs are selected by ROC curve analysis).

Age category (years)	<37.5		≥37.5	
	>5.7	≤5.7	>5.7	≤5.7
DHEAS category (*μ*mol/L)	Group 1	Group 2	Group 3	Group 4
	(*n* = 24)	(*n* = 45)	(*n* = 9)	(*n* = 51)
Patient characteristics				
Age (y)	34.0 ± 2.7^(3)(4)^	34.1 ± 1.8^(3)(4)^	39.7 ± 1.7^(1)(2)^	40.5 ± 1.9^(1)(2)^
BMI (kg/m^2^)	23.5 ± 3.8	23.0 ± 3.3^(3)(4)^	26.2 ± 5.0^(2)^	24.5 ± 3.7^(2)^
AFC	7.0 ± 4.0	7.4 ± 3.6	6.0 ± 3.0	5.8 ± 3.7
AMH (pmol/L)	3.2 ± 1.9	3.5 ± 1.8^(4)^	2.3 ± 1.5	2.6 ± 1.6^(2)^
DHEAS (*µ*mol/L)	7.0 ± 0.7^(2)(4)^	3.6 ± 1.2^(1)(3)^	6.9 ± 0.9^(2)(4)^	3.5 ± 1.4^(1)(3)^
FSH (IU/L)	9.0 ± 2.9	9.6 ± 8.3	9.1 ± 4.1	10.3 ± 6.9
LH (IU/L)	4.3 ± 1.8	4.6 ± 2.6	3.9 ± 1.1	5.0 ± 2.2
*E*_2_ (pmol/L)	192 ± 73	217 ± 103	248 ± 130	239 ± 153
*T* (nmol/L)	1.9 ± 0.6^(2)(4)^	1.2 ± 0.5^(1)(3)^	2.0 ± 1.9^(2)(4)^	1.3 ± 0.6^(1)(3)^
Treatment variables				
Duration of stimulation (d)	10.7 ± 1.3	10.3 ± 1.6	10.6 ± 3.0	10.7 ± 1.5
Total gonadotrophins used (IU)	3200 ± 392	2953 ± 643	3167 ± 901	3064 ± 633
Number of ICSI cycles, % (*n*/*N*)	73.9 (17/23)	72.2 (26/36)	71.4 (5/7)	76.2 (32/42)
IVF outcomes				
Number of oocytes retrieved	3.1 ± 1.4^(4)^	3.2 ± 2.4^(4)^	2.9 ± 1.7	2.0 ± 1.5^(1)(2)^
Number of oocytes eligible for fertilization	2.5 ± 0.8^(4)^	2.2 ± 1.1^(4)^	2.1	1.6^(1)(2)^
Number of embryos transferred	1.5 ± 1.1	1.3 ± 1.2	0.9 ± 1.1	1.0 ± 0.9
Implantation rate per embryo transferred (%)	32.4^(2)(4)^	8.6^(1)(4)^	12.5	3.8^(1)(2)^
Clinical pregnancy rate per cycle initiated (%)	37.5^(2)(4)^	8.9^(1)^	11.1	2.0^(1)^

Note: values are presented as mean ± SD. AFC: antral follicle count; AMH: anti-Müllerian hormone; BMI: body mass index Conversion formulas for the AMH, DHEAS, *E*_2_, and *T* values in mass units are as follows: ng/mL = pmol/L: 7.14, *μ*g/dL × 0.027 = *μ*mol/L, pg/mL × 3.67 = pmol/L, and ng/dL × 0.0347 = mmol/L, respectively. Fisher's exact test, Chi-quadrat test, and Kruskal-Wallis test with post-hoc analysis according to Conover were used for between-group comparison where appropriate. Superscripted numbers in parentheses denote ^(1)^ *P *< 0.05 compared with Group 1; ^(2)^ *P *< 0.05 compared with Group 2; ^(3)^ *P *< 0.05 compared with Group 3; ^(4)^ *P* < 0.05 compared with Group 4.
